# Cellular Senescence, a Novel Area of Investigation for Metastatic Diseases

**DOI:** 10.3390/cells12060860

**Published:** 2023-03-10

**Authors:** Francesca Faggioli, Michael C. Velarde, Christopher D. Wiley

**Affiliations:** 1 IRCCS Humanitas Research Hospital, Via Manzoni 56, Rozzano, 20089 Milan, Italy; 2 Istituto di Ricerca Genetica e Biomedica (IRGB-CNR) uos Milan, Via Fantoli 15/16, 20090 Milan, Italy; 3 Institute of Biology, College of Science, University of the Philippines Diliman, Quezon City PH 1101, Philippines; 4 Jean Mayer USDA Human Nutrition Research Center on Aging, Boston, MA 02111, USA; 5 School of Medicine, Tufts University, Boston, MA 02111, USA

**Keywords:** cellular senescence, metastasis, metabolic adaptation, invasion

## Abstract

Metastasis is a systemic condition and the major challenge among cancer types, as it can lead to multiorgan vulnerability. Recently, attention has been drawn to cellular senescence, a complex stress response condition, as a factor implicated in metastatic dissemination and outgrowth. Here, we examine the current knowledge of the features required for cells to invade and colonize secondary organs and how senescent cells can contribute to this process. First, we describe the role of senescence in placentation, itself an invasive process which has been linked to higher rates of invasive cancers. Second, we describe how senescent cells can contribute to metastatic dissemination and colonization. Third, we discuss several metabolic adaptations by which senescent cells could promote cancer survival along the metastatic journey. In conclusion, we posit that targeting cellular senescence may have a potential therapeutic efficacy to limit metastasis formation.

## 1. Introduction

The word “metastasis” is derived from the Greek words meta (meaning beyond) and stasis (indicating the basic concept of motion from one site to another). Indeed, metastasis development requires that cancer cells leave the primary cancer site and disseminate in the bloodstream until seeding to a permissive secondary site. From extensive research, we now know that metastasis underlies the capacity for physical movement, but also the epigenetic, transcriptional and secretory plasticity needed to survive [1]. Metastasis is the most lethal consequence of cancers, but only a few cancer cells survive the initial dissemination. This apparent vulnerability is counterbalanced by the proliferation of a tiny proportion of disseminated cancer cells (DCcs), which are difficult to target for therapeutic purposes [2].

Metastasis does not necessarily follow a linear model of progression. Some cancer cells most likely were ‘born to be bad’, wherein invasive and even metastatic potential is specified early, instead of acquiring deleterious genetic modification through a series of clonal expansions [3]. Regardless of cancer origin, DCcs need to finely tune the balance between cell proliferation and resting status, which is commonly known as “dormancy”, to evolve into a lethal form of metastasis. These features are theoretically intrinsic properties of cancer cells, but even in the first studies of Steven Paget’s seed and soil hypothesis, it was evident that the crosstalk with the microenvironment was of high importance for the success of distant organ colonization. Several studies report that cancer cells need to continuously adapt to changing environments during dissemination, including their metabolism to survive and seed new tumors, at the expense of other abilities [4].

**Figure 1:**
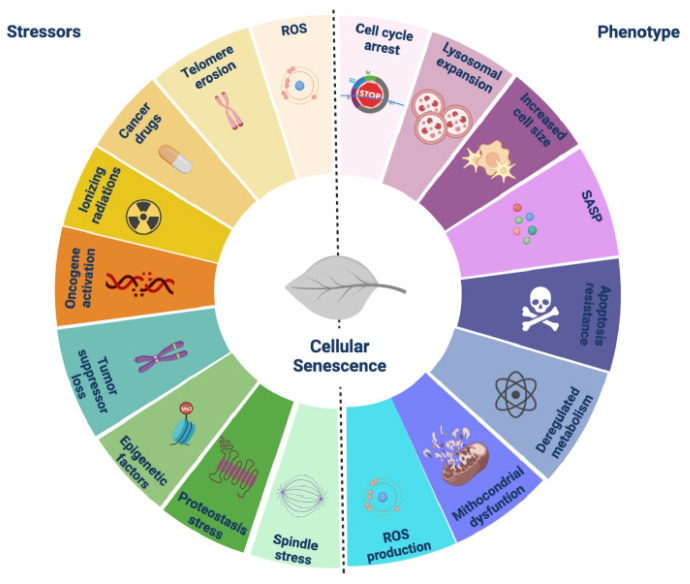
Senescence-inducing stimuli and related phenotypes.

Cellular senescence is a complex stress response. Stimuli of induction and common phenotypes are shown in Figure 1 and are extensively reviewed elsewhere [5]. The main features of senescent cells are a consequence of a dynamic remodeling in morphology, chromatin and cellular metabolism, which confer their typical flat large shape, loss of proliferation potential and inhibition of cellular apoptosis. Cellular senescence is also linked to cancer. As a stable cell cycle arrest, senescence is, in the short-term, tumor-suppressive. Indeed, the loss of proteins required for senescence, such as p53, p16^INK4a^ or Rb, is a common step in carcinogenesis in human tumors and animal models [6,7,8]. Senescence accumulates at high levels in pre-malignant lesions and at lower levels in invasive diseases [9,10,11], supporting its role as barrier in malignant progression. However, the percentage of senescent cells in a tumor may vary according to the cancer type and chemotherapy treatments, which also cause aging in cancer cells [12]. Senescent cells also drive inflammation through the secretion of a mélange of factors collectively referred to as the senescence-associated secretory phenotype (SASP), by which they can also manipulate tissue microenvironments; therefore, the activation of their secretome and the resulting beneficial or detrimental effects are context-dependent [8]. Nonetheless, targeting senescent cells has emerged as a new therapeutic approach to treat age-related diseases, and this treatment approach has also been extended for cancer. Indeed, cancer cells’ one-time senescence is more vulnerable to “senolytic” drugs, which target the anti-apoptotic response active in senescent cells. In this context, the removal of senescent cancer cells after chemotherapy treatment may represent a viable option to maximize the conventional therapy and prevent cancer relapse. In addition, there is an increased attention regarding the role of cellular senescence in metastasis. The pioneering work of Young Hwa Kim indicates that senescent cancer cells are actively involved in the invasion and metastasis of thyroid cancer [13]. More recently, Sugimoto’s group demonstrated that even in melanoma, senescent cells promote metastasis in a non-cell-autonomous manner [14]. Far from being an exception, these studies revealed a tight connection between cellular senescence and DCcs, which helps in understanding the role of senescent cells in mechanisms beyond metastasis progression and opens unprecedent opportunities for the development of ad hoc senolytics as novel therapeutic tools for metastatic diseases.

In this review, we discuss the growing evidence for the dynamic interplay between cellular senescence and the process of metastasis. We focus on features of senescent cells that are shared with metastatic cells and are known to promote metastasis progression. We also explore the cancer-metabolic traits required for cancer cells to survive and soil in secondary organs and their connection with cellular senescence. These traits may then be explored as targets for a new generation of therapeutic approaches.

## 2. Invasion: Lessons from Mammalian Placenta

The degradation of the tumor basement membrane is the first step in cancer cell invasion. DCs gain access to the vasculature initially, and then escape by extravasation and home toward a target organ following a chemokine gradient. The evidence to support the migratory properties of senescent cells comes from their recent detection in lymph nodes and lymphovascular channels in human thyroid cancer [13]. Since a considerable number of primary human cancers accumulate senescent cells at marginal zones, it is also most likely to expect senescent cell dissemination in other cancer types [13,15]. However, this feature still needs to be validated experimentally and the underlying molecular mechanism must be further explored.

Valuable clues to the role of senescent cells in cell metastasis can be inferred from placentation during embryonic development [16]. The placenta is a provisional fetal organ, which develops from the blastocyst after implantation, and it is involved in the physiological adaption of the mother to immunological acceptance, nourishment and support of the developing embryo. As the placenta develops, it invades surrounding organs, behaving in some ways like cancer [17]. The placenta generates from trophoblast, the outer layer surrounding blastocyst, which drives the attachment to the uterine epithelium and then differentiates by fusion in the syncythiotrophoblast. This structure is a multinucleated layer which does not show any proliferative activity but displays an invasive phenotype [18,19]. Indeed, the syncythiotrophoblast exhibits the characteristic of cellular senescence, including SA-β-gal activity and p16, p53 and SASP factors with invasive properties [20]. Because the syncytiotrophoblast invasion of maternal tissues is critical for placentation, failure of these cells to undergo cell senescence and elicit the SASP, including the expression of extracellular matrix-degrading enzymes such as MMP2 and MMP9, compromises mouse placental development, causing intrauterine growth restriction during pregnancy [21]. However, in the presence of a cohesin defect, the natural program of placental senescence is exacerbated by persistent DNA damage, resulting in a negative impact on embryo outcome, partly restored by the inhibition of cytokine signaling [22].

**Figure 2:**
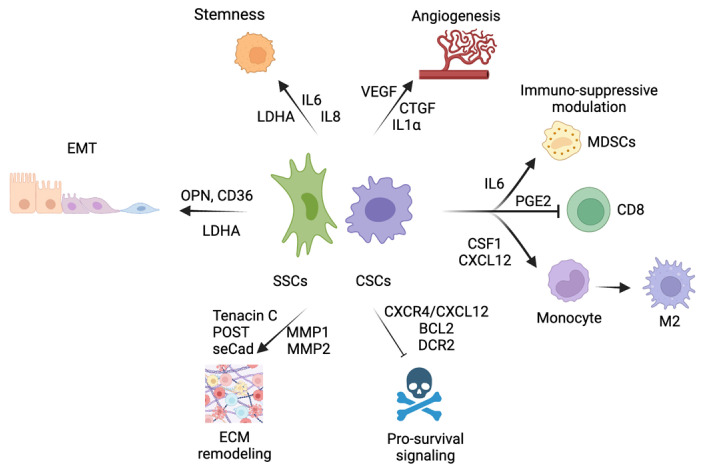
Summary of the factors secreted by senescent cancer cells and stromal senescent cells in each feature required for cell invasion. SSCs (senescent stromal cells); CSCs (cancer senescent cells); seCad (soluble E-cadherin).

Similar secreted cytokines with analogous functions have been described in cancer. Indeed, the IL-6 and IL-8 signaling pathways have shown to have both cell-autonomous and paracrine effects on stromal surroundings, playing a role in angiogenesis, epithelial to mesenchymal transition (EMT) and immune-suppressive responses in several cancers [23,24]. CCL5 is elevated in tumor-conditioned lymphatic endothelial cells and directs the dissemination of tumor cells into lungs and lymph nodes, promoting angiogenesis and colonization in distant organs [25]. CXCL1 is critical for premetastatic niche formation in the liver [26]. Therefore, it is not surprising that senescent cells similarly manipulate the microenvironment to promote the invasion of nearby tissues, such as the placenta. Indeed, in melanoma, soluble E-cadherin produced by senescent cells promoted ECM remodeling, increasing in vitro and in vivo the invasion properties of cancer cells. The elimination of senescent cells suppressed lung metastasis, indicating that the inhibition of senescence or related SASP can alter the metastatic potential in melanoma [14]. Considering the active involvement of proinflammatory cytokines in metastatic niche formation, it is possible that also for other types of cancers, senescent cancer cells could survive during dissemination and exploit their secretory phenotype to promote non-senescent cancer cell invasion in distant organs. In support of this hypothesis, we report below the characteristics needed for metastasis outgrowth (Figure 2).

## 3. Common Features Required for Cell Invasion and Colonization

### 3.1. Fusogenic Potential

Cell fusion is a physiological process in placentas and mammalian livers [27], bone [28] and muscles [29], where it contributes to the acquisition of a mature tissue phenotype. In cancer, cell fusion could combine properties of distinct cells to confer a functional advantage. Indeed, multiple experiments have shown that tumor cells become more aggressive after spontaneous fusion with cells of target organs, such as bone marrow cells, or acquire invading properties, perhaps by fusion with stroma or macrophages [30,31,32]. Hybrid cells have been shown to express several genes associated with tumor invasion and metastasis, such as SPARC, MCR1 and MET [32].

How cell fusion and cellular senescence are related in cancer remains unclear, although there is a common consensus that the fusion of normal and cancer cells can lead to cell cycle arrest [33,34]. The mutation of the tumor suppressor and senescence-effector p53 confer novel proliferating skills to hybrids, supporting a link between cell fusion and cellular senescence. For example, the overexpression of ERWVE1, the placental endogenous fusogen, and the measles virus (MV) in normal human fibroblasts and human alveolar adenocarcinoma cells (A549) results in the formation of syncitia with senescence features [20]. Many viruses evolve proteins that can prevent infected cells from undergoing senescence [35], suggesting that cellular senescence is a natural consequence of viral infection. Alternatively, senescence could prevent optimal viral replication, and therefore could be an important defense against viral infections. Indeed, the resulting polynucleated fused cells exhibit a senescence phenotype and the activation of p53- and p16–pRb main molecular effectors but also increased tumorigenicity assessed by colony assays. This dual activity may be explained by the pleiotropic function of the SASP.

ERVWE1 is expressed in cancer cells of multiple origins and mediates the fusion of cells in human tumors, including breast and colon cancer [36,37,38]. Even if every cell fusion event does not result in the induction of cellular senescence [39], it is likely that cellular senescence is a frequent outcome in tumoral hybrids. Indeed, the fusogenic potential of many tumors has been estimated at around 1%. [36,38,39,40]. Considering that the diameter of cancer cells spans from 15 to 25 μm, a metastatic tumor with a volume of 1 cm3 could contain at least one million of senescent hybrids. Notably, despite the oncogenic potential of cell fusion, most cases of cell fusion do not lead to cell transformation [33], suggesting that interaction with the tissue microenvironment is still a limiting or a promoting factor.

### 3.2. Polyploidy

Polyploidy is an important consequence of cell fusion, but can also occur by other processes such as endoduplication. Polyploidy is included in the developmental programs of many organs, including placenta, liver, heart and bone marrow [41,42,43,44]. Interestingly, in all cited areas, the acquisition of an extra amount of DNA coincides with growth restrictions, even though a direct connection between senescence features and ploidy increase is not yet conclusively demonstrated for each. In cancer, polyploidy is often a consequence of exposure to DNA damage or mitotic-checkpoint-targeting drugs [45]. The resulting polyploid tumor cells, referred to as Polyploid Giant Cancer Cells (PGCCs), have a large size, multiple nuclei, active metabolism and markers typical of therapy-induced senescence (TIS), including cell cycle arrest and secretion of tumor-promoting factors [46]. Not all PGCCs are β-gal positive, as described for MCF7 breast carcinoma cells after irradiation [47]. The authors explained this paradox by tracing simultaneous positivity for stemness and linked the absence of β-gal staining to an involvement in self-renewal [48]. Alternatively, Mirzayans et al. described active p53 as a determinant for the development of senescence in cancer cells rather than low β-gal activity in PGCCs. This result was shown in several human cell lines after ionizing radiation or chemotherapeutics [49].

PGCCs have been extensively identified in multiple cancers, including colon, melanoma, lung, pancreas, breast, ovarian, prostate, renal, thyroid and urinary bladder—and clinically detected in lymph nodes of metastatic prostate cancer [50]. Whole-Genome Doubling (WGD) was identified in 37% of solid tumors [51] and proposed as a marker of poor prognosis [52], suggesting that polyploidy in cancer cells can have a detrimental effect. Conversely, a protective role of polyploidy was identified in diethylnitrosamine (DEN) and high-fat diet models of liver carcinoma [53], so the effects of polyploidy are at least somewhat context-dependent.

### 3.3. EMT–MET Stemness

The transdifferentiation from an epithelial to mesenchymal state (EMT) and the inverse process, known as mesenchymal–epithelial transition (MET), are dynamically involved in cancer cell adaptation and survival [54]. The impact of full EMT or MET profiles on metastasis has been the center of an important debate in recent years, largely due to experimental limits in the ability to detect and follow EMT in clinical settings or with appropriate preclinical models [55]. EMT programs differ based on the inducing pathways, driver genes and cell populations [56]. Nevertheless, studies demonstrate that EMT is crucial for metastasis initiation and chemoresistance [57,58], transforming epithelial cells into invading cells, which disseminate and adapt to stress [59]. In contrast, tumors return to their epithelial state by MET during colonization [1,60,61], which appears to be crucially controlled by niche signals [62].

In aging, several reports describe the involvement of cellular senescence in promoting stemness and EMT features in an intrinsic manner. As stated above, senescent PGCCs often display EMT stem-like properties [63,64,65]. Those features are observed independently of polyploid status. Indeed, the overexpression of oncogenic H-RasG12V in normal mesothelioma cells or after pemetrexed treatment resulted in senescence induction and EMT with chemoresistance properties [66]. In acute lymphoblastic and myeloid leukemia, we described a new mechanism called senescent associated stemness (SAS), in which after DNA damage these cells developed senescence-associated stemness features, promoted by the activation of a Wnt embryonic development pathway [67]. When p53-dependent cell cycle arrest was blocked, these cells displayed higher tumor-initiating capacity than their senescent counterparts after transplantation in immune-competent recipient mice. Furthermore, the coexistence of the senescence marker p16Ink4a with markers of stemness and metastatic potential has been identified in patients of non-responsive breast cancer tumors after neoadjuvant chemotherapy [68]. These findings underly the intrinsic pro-tumoral nature of senescent cancer cells, which also develop EMT and stemness traits in humans [69,70]. Of note, this result is often achieved after chemotherapy, suggesting a secondary “dark side” to this type of clinical intervention.

In addition, stromal senescent cells can promote EMT and stemness through the SASP in pre-malignant or normal cells. Senescent fibroblasts or their conditioned medium stimulate premalignant epithelial cells to proliferate in culture and form tumors in mice [71]. In pre-malignant mammary or prostate epithelial models, cells become invasive and undergo malignant transformation [72]. These effects were largely due to interleukin IL-6 and IL-8 [62], and were later validated in rectal cancer and mesothelioma [66,70].

Osteopontin (OPN) was identified as one of the most highly elevated transcripts in senescent fibroblasts [69,73,74]. OPN promotes preneoplastic human keratinocyte cell growth both in vitro and in vivo [75]. Blocking OPN in breast cancer cells decreases the expression of SNAIL, SLUG and TWIST, indicating that OPN is critical for EMT and tumor metastasis even in a cell-autonomous manner. Additionally, OPN nuclear translocation induces MET and metastatic colonization in HCCLM3 human hepatocellular carcinoma cell lines after subcutaneous injection in athymic mice [76]. OPN translocation is driven by the SASP factor vascular endothelial growth factor (VEGF) from the microenvironment. The detection of nuclear OPN in clinical specimens of lung metastases supports the existence of this mechanism in human cancers [75]. Therefore, multiple SASP factors could interact synergistically to support tumor metastasis from different cellular sites along the metastatic journey.

Cancer-associated fibroblasts (CAFs) are known to have metastasis-promoting functions [77]. Intriguingly, CAFs frequently exhibit SASP-like factors and are enriched in senescent fibroblasts. CAFs have been shown to produce ECM niche components such as periostin (POSTN) and tenascin C [78,79], which are produced by senescent fibroblasts [80]. In addition, TGF-β released from colorectal cancer cells stimulates CAFs to secrete IL-11, which feeds back to tumor cells to activate STAT3 signaling, favoring the survival of metastatic cells in the liver [81]. IL11-induced secretome in primary cultures of human kidney, lung or skin fibroblasts comprises several SASP factors, including IL6, IL8 and CCL20, which are also important in the tumor microenvironment [82]. Even though the numbers of senescent CAF have not been identified in all contexts, experimental studies suggest that targeting senescent fibroblasts in this context has substantial consequences for disease relapse. Indeed, cancer therapy promotes the generation of senescent fibroblasts, which contribute to therapy resistance and increase cancer spread and relapse [83].

Senescent cancer cells can also induce metastatic activity in non-senescent surrounding cancer cells. Her2-dependent senescent MCF7 cells, when co-injected orthotopically with MDA-MB-231 epithelial cells in nude mice, promoted non-senescent cancer cell growth and invasion by their secretome [84]. Paracrine senescence can also involve endothelial cells and immune cells (see below). Together, these reports indicated that EMT and stemness are intrinsic properties of senescent cells that can also be induced in the surrounding microenvironment to promote the invasion of DCS and to set up a more permissive niche by SASPs.

### 3.4. Angiogenic and ECM Remodeling Properties

A key function of the pre-metastatic niche is to facilitate the landing of cancer cells, providing anchorage while circumventing anoikis and apoptosis [85]. Disseminating cancer cells benefit from angiogenesis and vascular leakiness, which can also have proinflammatory effects increasing the levels of chemoattractant factors, also increasing the recruitment of circulating tumor cells. The ECM is also important in metastatic seeding, as it provides enrichment in fibronectin, TGF-β, MMPs and other remodeling factors essential for permissive composition and stiffness [86,87,88]. All of these features are modulated by the SASP in primary tumors and by intrinsic resistance of senescent cells to apoptosis [89]. MMPs and factors related to matrix extracellular remodeling are active component of SASPs [90]. Following ionizing radiation, the co-culture of fibroblasts increased breast cancer cell invasiveness through the release of MMPs from senescent fibroblasts [91]. MMP-1 and MMP-2 secreted from senescent skin fibroblast also promote tumorigenic keratinocyte invasion by activating serine/threonin protein kinase PAR-1 [92]. The SASP also contains many pro-angiogenic factors (VEGF, GRO, CTGF, FN, laminin, PA, MMPs, PGE2, etc.) that establish a positive feedback loop with proinflammatory cells, promoting new vasculature, invasion and tumor progression [93]. Indeed, malignant epithelial cells co-injected with senescent fibroblasts have larger and greater numbers of blood vessels compared with controls. Conditioned medium from senescent fibroblasts enhances human umbilical vein endothelial cell invasion [74]. Connective tissue growth factor, CTGF, is another SASP component that facilitates LNCaP human prostate cancer progression by enhancing blood vessel formation [94]. Further, IL-1α, an endothelial surface adhesion molecule and a SASP factor, is involved in the mechanical contact and transient attachment of circulating cancer cells with microvasculature. This step elicits the trans-vascular penetration and consequent dissemination of cancer cells [95]. Thus, senescent cells are poised to support the development of new vasculature and extracellular remodeling during the metastatic seeding and anchorage of progressing tumors.

### 3.5. Immune-Suppressive Modulation

Tumor cells engage different immune cells to promote an immunosuppressive environment, which favors metastasis colonization. Among the different immune cell subtypes, myeloid-derived suppressor cells (MDSCs) exert a consistent immunosuppressive action in different organs, including liver, lung, bone and brain [96,97,98]. MDSCs secrete OPN, (see above), which interferes with CD8+ T cell proliferation, thus favoring lung metastasis. CXCR2 signaling in MDSCs and neurophils reduces CD3+ cell infiltration in liver metastasis, mimicking the immune-suppressive effect observed in the lung [99].

In a mouse model that develops premalignant prostatic lesions after the ablation of PTEN, a strong senescence tumoral response progresses to invasive adenocarcinoma [100]. This was linked to an immunosuppressive environment enriched in MDSCs cells and CXCL1/CXCL2, GM-CSF, M-CSF, IL10 and IL13 SASPs factors. Pharmacological inhibition of the immune-suppressive secretory arm of SASP favors an antitumoral microenvironment, which restored inflammatory immune cells and tumor resolution. In the same model, GR1+ cells secrete interleukin-1 receptor antagonist (ILRA), protecting a fraction of cancer cells from entering senescence [101]. The immunosuppressive nature of the SASP was also observed in a mouse model of obesity-induced hepatocellular carcinoma. Hepatic stellate cells become senescent after exposure to lipoteichoic acid (LTA) and deoxycholic acid, two obesity-induced gut microbial components. The resultant prostaglandin PGE2 production results in the attenuation of host antitumor immunity and HCC progression [102]. Finally, in colorectal cancer, senescent tumor cells can generate a CXCL12 gradient that inhibits CD8+ T cell chemotaxis by orchestrating CXCR4 plasma membrane receptor loss in vitro and in vivo. Senescent cancer cells also secreted colony-stimulating factor 1 (CSF1), which enhanced monocyte differentiation into M2 macrophages, with known inhibitory properties on CD8+ T cell activation [15]. While these data were collected on primary tumor models, this, in principle, can be expected to extend to secondary tissue niches. Importantly, the complexity of the SASP, which is cell type-, time-, and stressor-dependent, is a source of multiple possibilities for metastatic activation and has to be evaluated in the context of the model and disease studied [103].

### 3.6. Pro-Survival Intrinsic and External Anoikis Signals

Signals that prevent cell death are critical for metastatic cells to colonize new tissues [104]. Resistance to apoptosis is a common feature of many senescent cells [89]. Indeed, one of the earliest strategies for killing senescent cells was developed on the hypothesis that senescent cells were more sensitive to the inhibition of those pro-survival networks compared to non-senescent cells. Senescent cells upregulate the anti-apoptotic proteins BCL-W and BCL-XL, and genetic or pharmacological inhibition of BCL-W and BCL-XL selectively induces apoptosis in senescent cells [105,106,107]. The in vivo administration of ABT-737 eliminates senescent cells in both the lung and epidermis. Lipofuscin, commonly elevated in senescent cells, stimulates the expression of the anti-apoptotic factor Bcl-2, conferring resistance to apoptosis [108]. Another possible mechanism was attributed to the decoy death receptor DCR2, a cell surface receptor elevated in mouse senescent cells [109]. DCR2 protects senescent cells from immunity-mediated apoptosis, thus blocking the immune surveillance of senescent cells. This receptor is not conserved between mouse and human, but it is likely that human cells are able to develop cell-surface markers with immune-resistance activity, such as DCR2, which would explain their pro-survival tendency. Finally, CXCR4 and its ligand, CXCL12, can promote metastasis by preventing anoikis in cancer cells [13]. Indeed, senescent thyrocytes confer anoikosis resistance to co-cultured thyroid cancer cells via CXCR4-CXCL12. Functional CXCL12 knockdown or the CXCR4 antagonist AMD3100 decreases the survival of cancer cells. This latest discovery underlies the potential impact of senescent cells on the microenvironment and its paracrine survival action, which is extremely important in the context of cancer. An interesting mechanism of anoikis resistance in metastatic cells involves metabolic reprogramming to overcome ATP deficiency. Indeed, metastatic cancer cells secrete creatin kinase in the extracellular space to increase phosphocreatine, which is then imported and exploited as a source of ATP [110]. Thus, both senescent and metastatic cancer cells activate mechanisms that enable survival, allowing for cancers to spread to sites that may otherwise be inhospitable.

## 4. Metabolic Plasticity

Cancer cells often rely on glycolysis to synthesize the macromolecules required for continued cell division. To maintain this high level of glycolysis, most glucose-derived pyruvate is converted to lactate in the cytoplasm by lactate dehydrogenase (LDH), and secreted into the tumor microenvironment. This allows cancer cells to activate multiple biosynthetic pathways, which provide important building blocks for biomass synthesis and proliferation. This shift occurs even under oxygen-replete conditions with no defects in mitochondrial function, creating a pseudo-hypoxic state known as the Warburg effect [111]. However, cancer cells often adapt their metabolism during their metastatic journey [112], due to different nutrient and oxygen availability in “soil” tissues. Those tissue-specific metabolic features could also determine a priori their hospitability to circulating cancer cells, depending on the metabolic affinity with the primary organ. High levels of oxygen and glucose characterize brain and lung, which promote aerobic glycolysis or oxidative phosphorylation as an adaptive metabolism for energetic metastatic demand. Contrarily, the liver with low oxygen and fluctuating glucose levels relies on creatinine cycle activation to scavenge ATP or beta-oxidation [113]. Metabolic heterogeneity exists even within the same tumor due to variability in genetic alterations, epigenetic regulation, and transcriptional programs [114], further increasing complexity. Some metabolites are shared between primary cancer and metastasis, but others are specific for the metastatic site, and in some contexts, targeting only one of the nutrients that produce these molecules is sufficient to exhibit therapeutic efficacy in mouse models [115]. Thus, metabolism is a potential Achilles heel in the treatment of metastatic cells. Additionally, the large cell size and increased metabolism of polyploid tumor cells may represent a point of fragility specific to the polyploid subpopulation that could be exploited by metabolic targeted therapy.

The activation of glycolysis is also a feature of senescent cells [116,117,118]. Therapy-induced senescent lymphoma cells show increased glucose conversion to pyruvate, lactate and citrate. Senescent lymphoma cells lacking NF-κB, a master regulator of proinflammatory features of the SASP, have a muted SASP and more limited glucose consumption [117]. This suggests that most of the metabolic demand in senescent cells is linked to their secretory properties but also to the genetic alteration, like cancer cells. In support of this consideration, p53 and RB, the two tumor suppressors which mediate the major pathways of cellular senescence, drive different metabolic phenotypes in oncogene-induced senescence. While RB stimulates glycolysis, p53 favors the mitochondrial oxidation of pyruvate and fatty acids [119]. Similar to TIS, BRAF^V600E^ oncogene-induced senescent melanoma cells showed an increase in both glucose consumption and pyruvate metabolism [120].

Metabolites such as lactate, pyruvate, glutamine and lipids appear to be crucial metabolites in many steps of the metastatic cascade. The senescent program seems to be tightly connected to metabolism and metabolic stress. For simplicity, we summarized below the key findings which have an evident impact on senescence and metastasis. For a fully comprehensive perspective on metabolism in cellular senescence, we referred to recent reviews [121,122].

### 4.1. Pyruvate and Lactate

Pyruvate and lactate in cancer cells are both involved in EMT and ECM degradation, favoring migration. Lactate dehydrogenase A (LDHA) converts lactate in pyruvate, and its inhibition impairs invasion and migration in in vitro assays of renal cell carcinoma (RCC), pancreatic cancer and prostate cancer—and decreased metastasis in an orthotopic renal xenograft model [123,124,125]. High levels of the monocarboxylate transporter 1 (MCT1), which regulates lactate exchange from the intra- to extracellular space, are linked to a lower survival rate in patients with bladder cancer [126]. However, the radiation of human breast cancer cells induces MCT1 upregulation, lactate efflux, and the activation of NF-κB associated with senescence [127]. The MCT1-NF-κB axis promotes pro-invasive EMT in cervix squamous carcinoma, osteosarcoma and breast mammary carcinoma mouse models [128]. These findings suggest overlapping functions for pyruvate and lactate in the context of metastasis and cellular senescence.

### 4.2. NAD+

NAD+ is a cofactor for multi-redox pathways and a substrate for different signaling enzymes, and a key factor in tissue homeostasis [129]. Higher NAD+/NADH and NADP+/NADPH ratios are found in cancer cells compared to their non-transformed counterparts, suggesting the important role of NAD+ in their metabolic conversion [130]. Indeed, NAD+ is an essential cofactor for GAPDH activity, and lactate production from pyruvate converts NADH into NAD+. The upregulation of the NAD+ salvage pathway, the main source of NAD+ in most cells, is a common feature in many cancer cells, and nicotinamide phosphoribosyl-transferase (NAMPT), the rate-limiting enzyme of this pathway, is elevated in several human tumors including gastric, glioma and colorectal cancer. In many tumors, NAMPT expression correlates with worse prognosis and metastasis [129]. Furthermore, NADH can be a cancer stem cell marker [131].

The relationship between NAD+ and senescence is complex. Oncogene-induced senescent human fibroblasts accumulate high levels of intracellular NAD+ and NAMPT, which promote parts of the SASP through the activation of NF-κB [132]. The inhibition of NAMPT also promotes senescence, but without many segments of the SASP [133]. Senescent cells can also promote NAD+ decline in aged tissue, through macrophage activation [134]. Aged liver and visceral white adipose tissues accumulate inflammatory M1-like macrophages with enhanced CD38 NADase activity, thereby reducing NAD+ levels. Senescent cells within those tissues enhance CD38 overexpression in macrophages through the SASP. Together, these data suggest that NAD+ depletion may limit tumorigenesis by limiting both glycolytic activation and the SASP, but potentially at the cost of driving age-related conditions.

### 4.3. Lipids

Lipids are the primary building blocks of cellular membranes, a major mechanism of energy storage, and the source of multiple cellular signals. Aside from essential fatty acids, most can be synthetized de novo, but all can be taken up from the extracellular compartment [135]. Lipids have been functionally implicated in several steps of the metastatic cascade, through distinct mechanisms. First, the overexpression of transmembrane receptors for fatty acid import is a common trait of cancer cells with increased invasiveness and migratory properties. Among these, CD36 is used as an early diagnostic biomarker for metastatic cancers. Metastasis-initiating cells express CD36, and blocking this receptor inhibits metastasis formation in human oral carcinoma and other human cancer types [136]. Further, a study of over 2500 cases of different types of cancers stratified in metastatic versus primary tumors revealed that the CD36 gene was frequently amplified in metastatic groups and, similarly, poor survival rates correlate with CD36 high-copy numbers [137]. CD36-associated fatty acid uptake is also able to promote EMT in hepatocellular carcinoma cell lines [138], whereas in cervical cancer, the pro-metastatic effect of CD36 was synergistic with TGFβ-induced EMT in cell lines [139]. CD36 is rapidly upregulated in multiple cell types in response to replicative, oncogenic and chemical senescence-inducing stimuli [140]. In senescent cells, CD36 is activated by amyloid beta proteins instead of fatty acids themselves. This interaction stimulates NF-κB-dependent cytokine and chemokine production, leading to senescence onset. Therefore, the CD36 receptor is actively involved in SASP production. While the CD36 amyloid interaction was not evaluated in the context of cancer, it is tempting to speculate its involvement in the invasiveness of CD36+ cancer cells.

Second, de novo fatty acid synthesis contributes to the invasion, migration capacity and colonization of cancer cells. Accordingly, fatty acid synthase (FASN) overexpression increases the peritoneal metastasis of ovarian cancers in mice, and promotes cellular colony formation and metastatic ability in vitro [141]. The inhibition of FASN with the compound orlistate impairs melanoma-induced metastases and angiogenesis in mice [142], and the silencing of FASN attenuates CD44 expression-induced signaling and metastasis formation in colorectal cancer mouse models [143]. FASN was similarly elevated in senescent cells and required for both the cell cycle arrest and SASP production in oncogene-induced senescence [144].

Third, fatty acids can be further processed in the cell; the resulting metabolites are involved not only in metastasis but also in senescence [145], suggesting that saturated fatty acids might be an important substrate for energy production in senescent cells and migratory properties in cancer cells. Indeed, senescent cells can store lipids in the form of triglycerides with incorporated free polyunsaturated fatty acid (PUFA) [146]. PUFA can be further metabolized into oxylipins, which have proinflammatory properties and senescence-regulatory properties [147]. The overexpression of 5-Lipoxygenase (5-LOX), which is known to promote cancer [148], also drives senescence-like growth arrest via a p53/p21-dependent pathway [149]. In cancer, PUFA are known to induce proliferation, apoptosis and angiogenesis [150]. However, their role in metastasis is still in its infancy due to the lack of experimental models which adequately recapitulate the complexity of human cancer, but recently, the intake of ω-6 PUFAs in the diet was shown to enhance the malignancy of tumor cells by the upregulation of pro-tumoral oxylipins (PGs, HETEs, DiHETrEs and HODEs) in a murine model of pulmonary squamous cell carcinoma, increasing proliferation, angiogenesis and molecular aggressiveness [151]. Increased oxylipin metabolism was also proposed as a marker for the evaluation of early stage of breast cancer [152]. Taken together, those reports highlight the need for future investigations on the senescence–metastasis axis.

## 5. Conclusions and Future Directions

**Figure 3:**
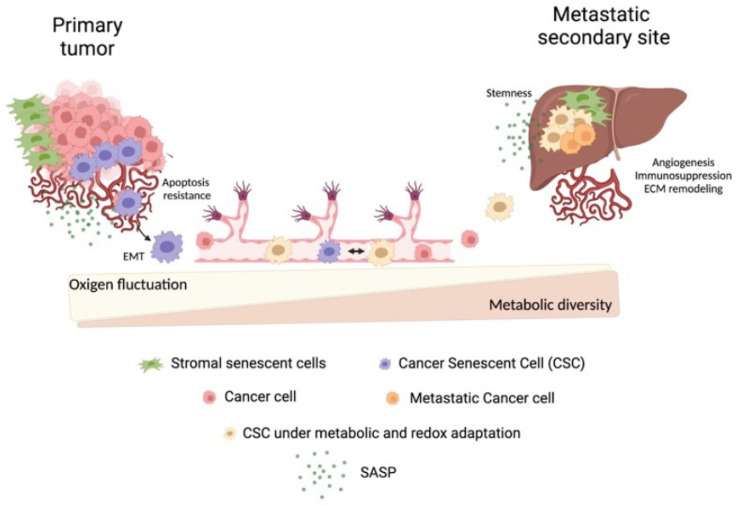
Summary of the intrinsic and extrinsic plasticity of senescent cells.

Here, we outline the intrinsic and extrinsic plasticity of senescent cells, both as cancer cells and as part of the stroma, supporting all the steps of metastasis development, from invasion to colonization (Figure 3). The extent to which cellular senescence in extra-embryonic contexts recapitulates placenta in cytokine secretion, fusogenic potential, polyploidy and stemness reveals their potential impact on metastasis. Cellular senescence is a stress response, but also a critical step in driving survival advantages in cancer. SASPs evoke several features related to metastasis progression and colonization, which can target cancer cells directly, but also niche components and immune cells, introducing multiple pathways for promoting tumor development. Senescent cells are metabolically active, but also metabolically plastic, and metabolic adaptation is a major rate-limiting step in the metastasis cascade because it defines the metabolic vulnerabilities of cancer cells. Despite this evident connection, many important biological questions remain unanswered. Do senescent cancer cells migrate from primary site to distant organs along with proliferative cancer cells? Are migrating senescent cancer cells heterogeneous in their transcriptional program and secretory production? Do senescent cancer cells undergo metabolic reprogramming and in what contexts? Do senescent cancer cells secrete metabolites that alter the ability of cancer cells to invade soil tissues? These questions will likely be addressed in coming years by emerging new technological approaches, including spatial transcriptomics at a single-cell resolution and the use of artificial intelligence on datasets from human specimens. This reflects the level of complexity of the role played by cellular senescence in the context of metastasis. Importantly, some metabolites are shared between primary cancer and metastasis, but others are specific to metastatic sites. Findings show that targeting single nutrients that produce these molecules is sufficient to exhibit therapeutic efficacy in mouse models, suggesting that metabolism is likely an Achilles heel in the treatment of metastatic cells. However, further studies are required to assess the functional relevance and significance of metabolic shifts in senescent cancer cells. For polyploid cancer cells, targeting senescence indispensable metabolites could be a new key for the intervention of aging-associated and age-related pathologies, including metastasis. These approaches will facilitate deep insights into the complex biological phenomenon of human metastasis and lead to a better understanding of the heterogeneity of cellular senescence, with the ultimate goal of developing more precise interventions for metastatic cancer.

The progression to deep invasion and metastasis may be fueled by SSCs at the primary and secondary site by paracrine SASP signaling. In the first case, SASP can lead to neo-angiogenesis and confer EMT- and stem-like properties to cancer cells, while in the soil organs, SASP can contribute to establish a permissive niche, being involved in ECM remodeling, immunosuppression and angiogenesis. CSC can detach from the primary tumor and enter in the circulation, in which they survive through their intrinsic resistance to apoptotic signaling and invasive properties of the EMT phenotype. During the journey, senescent cells can undergo a multi-step process of metabolic and environmental adaptation, which ultimately leads to their soil into the metastatic organ. At this site, CSCs could boost non-senescent cancer cells to a more aggressive stem-like state.

Fortunately, the contribution of senescence and the SASP to metastatic invasion offers hope for future therapeutic intervention [153]. This could come in two ways: (1) preventing the metastasis-promoting effects of senescent cells, or (2) eliminating senescent cells so that they cannot promote metastasis. Molecules that inhibit or restrain the SASP, such as rapamycin or metformin, have been shown to prevent cancer progression [154,155,156]. Molecules that selectively kill senescent cells, called “senolytics”, also show promise as cancer therapeutics. Indeed, one strategy that has been successfully employed in animals it to first render cancer cells senescent using chemotherapeutics [83], PARP inhibitors [157] or CDC7 inhibitors [158], followed by the elimination of these induced senescent cells using senolytic compounds. This “one-two-punch” approach may improve treatment options where other strategies fail [159]. Senescent cancer cells are antitumoral in the short term, while chronic exposure to the SASP leads to cancer recurrence and prolonged-therapy side effects. How do we select the deleterious senescent cells against the beneficial ones? To avoid the context-dependency issue of senescence-opposing effects, senolytic chimeric antigen receptor (CAR) T cell therapy could be used to selectively target only pathogenic senescent cells. Chimeric receptors that recognize cell-surface proteins specifically upregulated in senescence, such as uPAR-specific CAR T cells, effectively eliminate senescent cells in a lung cancer mouse model [160]. A less cost-effective strategy would be to generate antibody–drug conjugates that combine monoclonal antibodies specific to senescence-surface antigens with toxins [161].

Thus, while many questions currently remain unanswered, the need to dissect senescence heterogeneity in human cancers has become evident. Applying this knowledge holds great potential for opening new avenues in more effective cancer therapies.
